# Dexamethasone Provides Effective Immunosuppression for Improved Survival of Retinal Organoids after Epiretinal Transplantation

**DOI:** 10.1155/2019/7148032

**Published:** 2019-07-25

**Authors:** Bikun Xian, Ziming Luo, Kaijing Li, Kang Li, Mingjun Tang, Runcai Yang, Shoutao Lu, Haijun Zhang, Jian Ge

**Affiliations:** ^1^State Key Laboratory of Ophthalmology, Zhongshan Ophthalmic Center, Sun Yat-sen University, Guangzhou, Guangdong 510060, China; ^2^Bai Duoan Medical Equipment Company, Qihe Economic Development Zone, Qihe, Dezhou, Shandong 251100, China

## Abstract

We investigated the efficacy of the immunosuppressants rapamycin (RAP) and dexamethasone (DEX) in improving the survival of retinal organoids after epiretinal transplantation. We first compared the immunosuppressive abilities of DEX and RAP in activated microglia in an *in vitro* setting. Following this, we used immunofluorescence, real-time polymerase chain reaction, and flow cytometry to investigate the effects of DEX and RAP on cells in the retinal organoids. Retinal organoids were then seeded onto poly(lactic-co-glycolic) acid (PLGA) scaffolds and implanted into rhesus monkey eyes (including a healthy individual and three monkeys with chronic ocular hypertension (OHT) induction) and subjected to different post-operative immunosuppressant treatments; 8 weeks after the experiment, histological examinations were carried out to assess the success of the different treatments. Our *in vitro* experiments indicated that both DEX and RAP treatments were equally effective in suppressing microglial activity. Although both immunosuppressants altered the morphologies of cells in the retinal organoids and caused a slight decrease in the differentiation of cells into retinal ganglion cells, the organoid cells retained their capacity to grow and differentiate into retinal tissues. Our *in vivo* experiments indicate that the retinal organoid can survive and differentiate into retinal tissues in a healthy rhesus monkey eye without immunosuppressive treatment. However, the survival and differentiation of these organoids in OHT eyes was successful only with the DEX treatment. RAP treatment was ineffective in preventing immunological rejection, and the retinal organoid failed to survive until the end of 8 weeks. DEX is likely a promising immunosuppressant to enhance the survival of epiretinal implants.

## 1. Introduction

Glaucomatous optic neuropathy is one of the leading causes of blindness [1]. Although regulating intraocular pressure (IOP) is currently considered the most effective method to control glaucoma-induced blindness, the damage to retinal ganglion cells (RGCs) due to glaucoma is generally considered irreversible. To restore vision in such cases, damaged RGCs must be restored, which is possible only with stem-cell therapy [2, 3]. Owing to the anatomical and pathophysiological characteristics of glaucoma and other inner retinal diseases, epiretinal or intravitreal transplantation procedures have been found to be more successful than subretinal transplantation procedures [4]. Furthermore, unlike rodent eyes, human eyes have a smaller crystalline lens and larger vitreous cavities; because of this, cell-sheet transplants have a higher chance of success, in terms of integration into human eyes, than do cell-suspension injections [5].

Our research team has previously established a new surgical procedure for transplanting retinal organoids as sheets into rhesus monkey eyes [6]. However, although this procedure was successful for the placement and long-term fixation of an epiretinal transplant, the presence of pathological lesions at the transplantation site and the inevitable injuries inflicted during surgery might activate innate immunocytes and impede the survival and integration of grafted cells [7]. Even though this problem can be surmounted by autologous cell reprogramming, this process is highly labor-intensive and time-consuming, and in addition, directional differentiation for autotransplantation is also difficult to achieve. Therefore, immunosuppressive treatment might be a better option for such transplantation procedures.

Dexamethasone (DEX), which is a synthetic corticosteroid, is a common clinical immunosuppressant; despite its short half-life (a few hours) in the vitreous body [8], the intraocular application of DEX is usually used for the treatment of bacterial endophthalmitis [9] and macular edema [10]. Rapamycin (RAP) is a relatively new immunosuppressant often used to prevent the rejection of newly transplanted organs [11]. RAP is also used to treat autoimmune uveitis and is known to have an intraocular half-life of ~7 days [12]. Furthermore, RAP has also been shown to exert neural protective effects [13], which has generated great interest for its use as an immunosuppressant for transplants in cases involving glaucoma-induced eye conditions. In this study, we compared the effects of DEX and RAP on microglial cells and retinal organoids, both *in vitro* and *in vivo*, to determine which is effective in protecting and prolonging the survival of epiretinal transplants.

## 2. Materials and Methods

### 2.1. Immunosuppressive Effects of Dexamethasone and Rapamycin on Microglial Cells *In Vitro*

The microglial cell line BV2 (Shanghai Cell Bank of Academia Sinica, Shanghai) was maintained in Dulbecco's modified Eagle's medium with 10% fetal bovine serum. For the Cell Counting Kit-8 (CCK8) test (Dojindo, Japan), BV2 cells were seeded in 96-well plates (Corning, USA), cultured overnight, and activated by exposure to lipopolysaccharide (LPS; L288, Sigma-Aldrich, USA) for 1 h. The cells were then exposed to RAP (20 *μ*g/ml, S1039, Selleck, USA) or DEX (170 *μ*g/ml, Tianjin Jinyao Company, China) for 24 h; the cells in the positive control group for this experiment were treated without immunosuppressants and the negative control was the culture medium only. Following this, the cells were incubated in CCK8 reagent (10% *v*/*v*) for 2 h. The absorbance of the supernatant was measured at 450 nm using a spectrophotometer (Synergy H1, BioTek, USA). We also carried out enzyme-linked immunosorbent assays (ELISA; 70-EK1822, MultiSciences, China) to measure levels of tumor necrosis factor alpha (TNF-*α*) and the Griess assay (G2930, Promega Corporation, USA) to measure levels of NO produced by the microglia. After activation by LPS and treatment with DEX or RAP for 24 h, the supernatant was collected. For ELISA, the diluted supernatants were added to the microplate and the positive and negative controls were standard substances provided by the manufacturer and fresh medium, respectively. The antibody, streptavidin-horseradish peroxidase (HRP), and substrate solution were added and incubated with the reaction sequentially. Then, the stop solution was added, and the absorbance was measured at 450 nm and corrected at 570 nm using a spectrophotometer. For the Griess assay, the supernatants were reacted with sulfanilamide solution and N-1-napthylethylenediamine dihydrochloride solution. Positive and negative controls were nitrite standard and fresh medium, respectively. The absorbance was measured at 520 nm using a spectrophotometer.

### 2.2. Generation and Character of Retinal Organoids

Two human-induced pluripotent stem cell (hiPSC) lines, the BC1-GFP cell line (gifted by Prof. Linzhao Cheng, Johns Hopkins University School of Medicine) and the SB cell line (CA4002106, Cellapy, China), were used to generate retinal organoids. The hiPSCs were maintained in a feeder-free system in mTeSR1 medium (STEMCELL Technologies, Canada). They were then induced to differentiate into retinal organoids using the protocol published by Zhong et al. and Luo et al. [14, 15]. Briefly, hiPSCs were maintained in suspension until they formed embryoid bodies (EBs), following which, the EBs were induced to form retinal organoids after being sequentially cultured in N2 (17502-048, Gibco) and B27 (12587-010, Gibco) supplements.

After 30-45 days of induction, some retinal organoids were harvested and fixed in 4% paraformaldehyde (Sigma-Aldrich) for 30 minutes, then dehydrated using graded sucrose (Sigma-Aldrich) and embedded in optimal cutting temperature compound (O.C.T., SAKURA, Japan). And retinal organoids were frozen and sectioned via cryotomy (Leica, Germany). Some retinal organoids were attached on coverslips (Jieli Biotechnology Co. Ltd., China) or culture plates (Corning) coated with Matrigel (354277, Corning) and cultured with either RAP (20 *μ*g/ml) or DEX (170 *μ*g/ml).

### 2.3. Flow Cytometry

After 30-45 days of induction, retinal organoids were cultured adherently with either RAP (20 *μ*g/ml) or DEX (170 *μ*g/ml) for 2 weeks; positive controls were treated without immunosuppressants. The tissues were then digested into single cells using an Accutase™ Cell Dissociation Reagent (A1110501, Gibco) as per the manufacturer's instructions. The cell suspensions obtained were incubated with APC mouse anti-human CD90 (561971, BD Biosciences, USA) for 25 min. Flow cytometry was carried out using an LSRFortessa flow cytometer (BD Biosciences), and the data obtained were analyzed using the FlowJo software (Tree Star Inc., USA). All experiments were replicated three times.

### 2.4. Reverse Transcription Polymerase Chain Reaction (PCR) and Real-Time PCR (RT-PCR)

Total RNA from retinal organoid cells exposed to DEX or RAP for 2 or 4 weeks, as well as unexposed cells, were extracted using TRIzol reagent (T9424, Sigma-Aldrich), and cDNA was generated using a PrimeScript RT Master Kit (RR036A Takara Bio, China) according to the manufacturer's instructions. RT-PCR was performed using a LightCycler 480 SYBR Green I Master (4887352001–1, Roche, Switzerland) on a LightCycler 480II system (Roche). Primers for the genes *Atoh7*, *Islet1*, *Brn3b*, *TUBB3*, *NEFL*, *MAP2*, *PAX6*, *CHX10*, *CRX*, and *β-actin* (Table 1) were synthesized by Invitrogen Inc. All reactions were performed in triplicate. Gene expression levels were calculated using the 2−*ΔΔ*Ct method and normalized to those of *β-actin* that was used as an internal control.

### 2.5. Experimental Animals and Establishment of Chronic Ocular Hypertension (OHT)

This research project was approved by the Medical Ethics Committee of Zhongshan Ophthalmic Center, Sun Yet-sen University, Guangzhou, China. All procedures were performed in accordance with the Association for Research in Vision and Ophthalmology (ARVO) Statement for the Use of Animals in Ophthalmic and Vision Research and approved by the Institutional Animal Ethics Committee (Animal Welfare Assurance No. 2018-003). Four male rhesus monkeys aged 3–5 years were used in this study; three of these monkeys were randomly chosen for the induction of chronic OHT, according to a protocol previously published by Yan et al. [16]. Briefly, monkeys were anesthetized via the intramuscular injection of 3% *w*/*v* sodium pentobarbital (0.5 ml/kg, Sigma-Aldrich) and 2% *w*/*v* xylazine hydrochloride (0.1 ml/kg, Su-Mian-Xin II, Shengda Animal Drug Company, China), then 1% pilocarpine was used to induce miosis. Following this, a gonioscope (Volk, USA) was gently placed on the cornea, and a 360° photocoagulation was performed on a functional trabecular meshwork (Novus Varia, Lumenis, USA). OHT was sustained for 3 months by maintaining the IOP at >20 mmHg, by which time, the treated eyes had suffered retinal nerve fiber layer damage. These monkeys were then prepared for transplantation surgery.

### 2.6. Transplantation of Retinal Organoid-Poly(Lactic-Co-Glycolic) (PLGA) Composite and Post-Surgical Immunosuppressive Treatment

The retinal transplantation surgeries were performed on three OHT eyes and one healthy eye according to the protocol described by Luo et al. [6]. All surgical procedures were performed by the same surgeon. Briefly, to make a retinal organoid sheet, retinal organoids were attached to a Matrigel-coated PLGA (Bai Duoan Medical Equipment Company, China) scaffold 5 days before the surgery. Anesthesia was induced to the monkeys as before, then a standard 23G pars plana vitrectomy (Accurus400VS, Alcon, USA) was performed. Following this, one of the incisions was enlarged to 18G, and the retinal organoid-PLGA sheet was inserted into the eye through this incision; two retinal tacks were used to fix the sheet onto the retina.

The three post-operative immunosuppressive treatments including intravitreal (IVT) injections of RAP (90 *μ*g/eye/week), IVT OZURDEX® (single dose, dexamethasone IVT implant, Allergan, Ireland), and sham treatment (IVT injection of 100 *μ*l of phosphate-buffered saline (PBS)/eye/week) were administered after the incisions were sutured. The healthy eye transplanted with the retinal organoid-PLGA sheet was treated with IVT injections of 100 *μ*l of PBS/eye/week.

To evaluate the survival of the retinal organoid-PLGA sheet, and any immune reaction against the transplant, fundus photography (TCR-50DX, Topcon, Japan, and VET2, Optomed Oy, Finland) and optical coherence tomography (OCT, SPECTRALIS OCT, Heidelberg Engineering, UK) were performed every week after the surgeries.

### 2.7. Immunofluorescence and Histology

Eight weeks after the surgeries, the monkeys were euthanized with pentobarbital and Su-Mian-Xin II, then the surgically transplanted eyeballs were harvested and fixed in 4% paraformaldehyde (Sigma-Aldrich) overnight. The eyeball tissues were dissected to obtain 10 mm × 10 mm sections containing the transplants, which were frozen and sectioned via cryotomy (Leica). The cryosections and coverslips with retinal organoids were analyzed using immunofluorescence and hematoxylin and eosin staining as per the procedure described by Lu et al. [17]. Antibodies against the following markers were used as primary antibodies for immunofluorescence microscopy at the dilutions indicated in parentheses: Ki67 (1 : 100, A11390, ABclonal, China), CHX10 (1 : 200, AB9016, Millipore, Germany), Brn3 (1 : 200, SC-6026X, Santa Cruz, USA), Islet1 (1 : 20, BM44446, Boster, China), HuD (1 : 100, SC-48421, Santa Cruz), RBPMS (1 : 100, ab152101, Abcam), *β*III-tubulin (1 : 200, ab7751, Abcam), neurofilament light polypeptide (NEFL; 1 : 100, A0257, ABclonal), microtubule-associated protein 2 (MAP2, 1 : 100, BM1243, Boster), vimentin (1 : 50, BM4029, Boster), glial fibrillary acidic protein (GFAP; 1 : 500, 3670S, Cell Signaling Technology, USA), CD68 (1 : 200, ab201340, Abcam), Iba1 (1 : 200, ab5076, Abcam), and SC121 (1 : 500, Y40410, Takara Bio). The secondary antibodies used were Alexa Fluor-488- and Alexa Fluor-555-conjugated anti-mouse, rabbit, or goat antibodies (1 : 500; A21202, A21206, A31570, A31572, A21432, Gibco). Cell nuclei were stained with 4′,6-diamidino-2-phenylindole (DAPI, AR1177, Boster). Cells were viewed under an LSM 510 confocal microscope (Carl Zeiss AG, Germany) and a fluorescence microscope (Olympus, Shinjuku, Japan).

### 2.8. Statistical Analysis

All statistical analyses were performed using the SPSS 20.0 software (IBM, USA). Comparisons between groups were carried out using Student's *t*-tests and one-way ANOVAs followed by Bonferroni corrections. Results with *P* < 0.05 were considered statistically significant.

## 3. Results

### 3.1. Dexamethasone and Rapamycin Can Suppress Microglial Division and Function *In Vitro*

The effects of DEX and RAP on the division and function of BV2 cells activated by LPS are summarized in Table 2. As compared to that in the positive control, activated BV2 cells treated with RAP and DEX contained 57.22 ± 0.18% and 48.74 ± 0.04% fewer microglial cell populations, indicating that both immunosuppressants were capable of inhibiting cell division in activated microglia. Our results also indicated that RAP and DEX could inhibit microglial function by inhibiting the production of TNF-*α* and reactive oxygen species (ROS; measured based on the concentration of NO produced). TNF-*α* levels produced by RAP- and DEX-treated cells were significantly lower (437.74 ± 102.13 pg/ml and 605.51 ± 248.42 pg/ml, respectively) than those in the control group (2247.79 ± 257.57 pg/ml; *P* < 0.05). Similarly, ROS levels produced by RAP- and DEX-treated cells were significantly lower (14.89 ± 2.10 *μ*M and 20.46 ± 3.11 *μ*M, respectively) than those in the control group (39.70 ± 1.76 *μ*M, *P* < 0.05).

### 3.2. Changes in the Morphologies of Retinal Organoids in Response to Immunosuppressant Treatment

**Figure 1:**
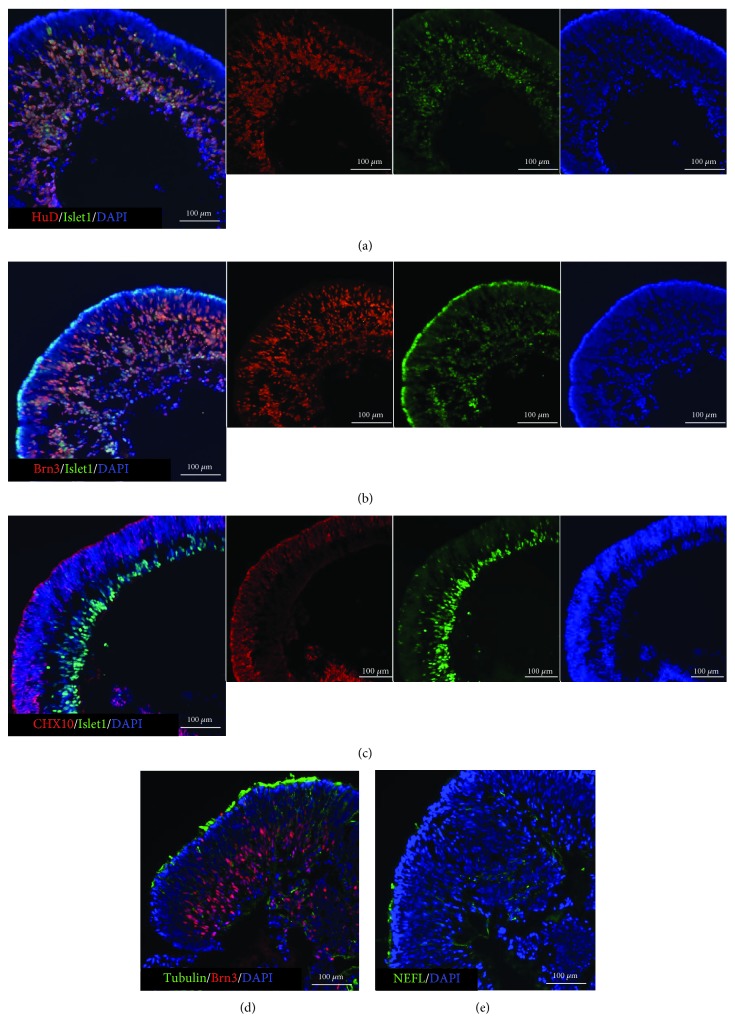
Characteristics of retinal organoids after 30-45 days of induction. The frozen sections of retinal organoids were identified by immunofluorescence staining. (a, b) At this time, most cells of the organoids were differentiating towards RGCs; they were Islet1-, Brn3- and HuD-positive. (c) But some cells outside were still retinal progenitors; they were CHX10-positive. (d, e) And in suspension cultivation, little axons were developed.

Retinal organoids in suspension culture were biconcave disc-shaped. At 30-45 days of induction, most cells from organoids differentiated into RGCs an they expressed RGC markers such as Brn3, Islet1, and HuD [18–20] (Figures 1(a) and 1(b)). In the early stage, RGCs appear first inside the organoids (Islet1-positive), and cells outside still expressed the retinal progenitor marker CHX10 (Figure 1(c)). When retinal organoids were in suspension cultivation, only little axons were seen in them (Figures 1(d) and 1(e), tubulin- and NEFL-positive).

**Figure 2:**
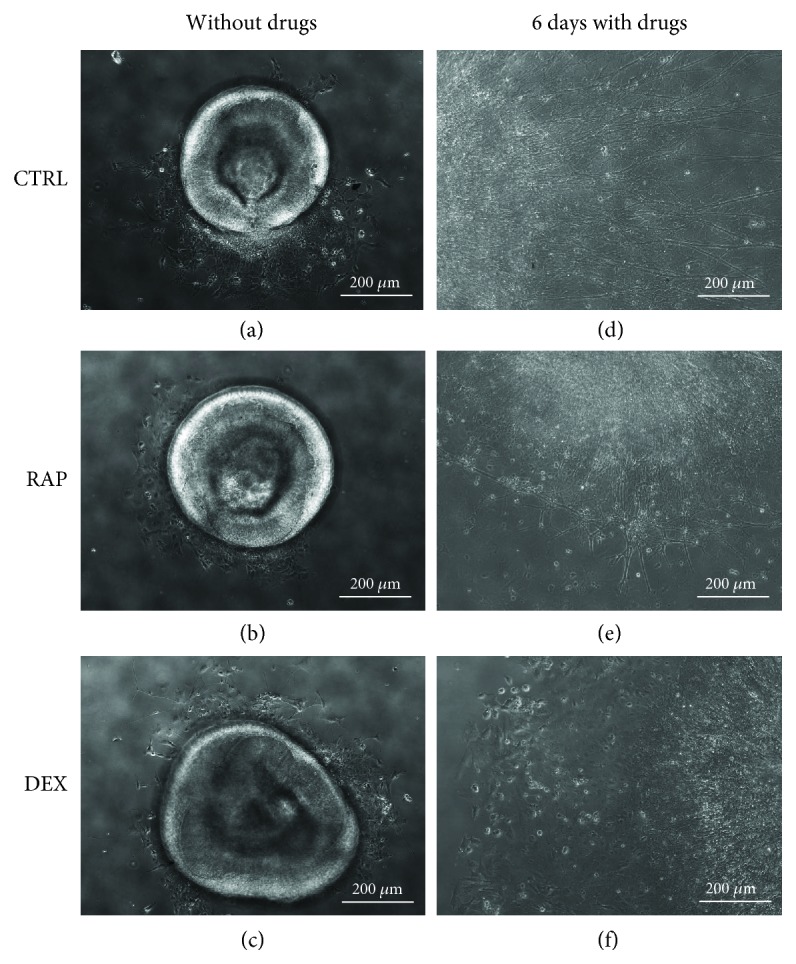
Morphology of retinal organoids exposed to dexamethasone (DEX) and rapamycin (RAP). Three-dimensional organoids shaped like biconcave discs were derived from human-induced pluripotent stem cells (hiPSCs). (a–c) After 4 days of adherent culture, although the organoids maintained their shapes, some cells were observed to migrate out from the organoids. Six days after culture with or without immunosuppressants, the morphologies of the retinal organoids changed. (d) Without drugs, the axons of the organoids were long, strong, and distributed radially; cells were also observed to have migrated farther away from the organoid. (e) With RAP treatment, axons were much shorter than those of cells in the control group; in addition, the axons tended to intertwine with each other at the edge of the organoid. (f) In the DEX-treatment group, long axons were very few and thin. CTRL: control group; RAP: rapamycin-treatment group; DEX: dexamethasone-treatment group.

**Figure 3:**
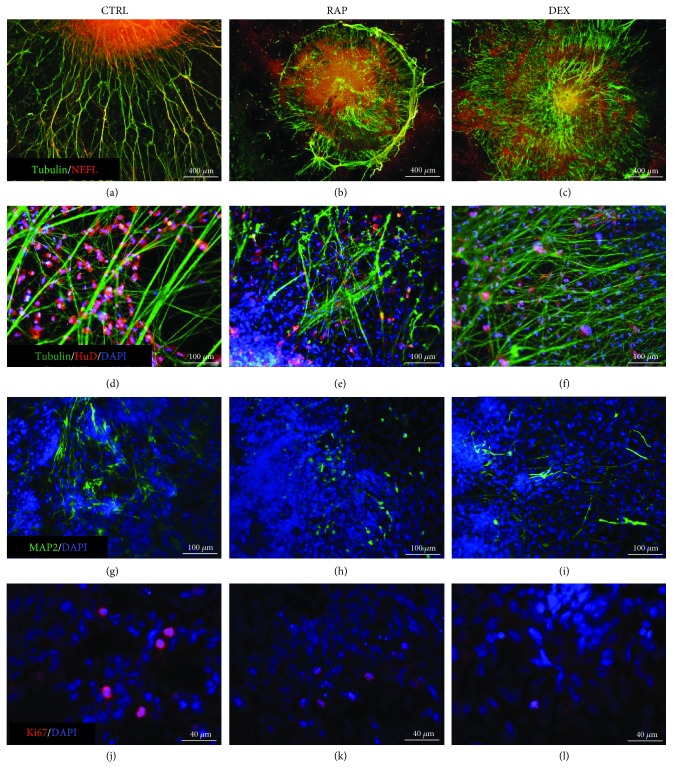
Immunofluorescence microscopy to examine the morphologies of cells from retinal organoids after exposure to immunosuppressants (a) Axons in the control group were long and strong and most expressed tubulin and NEFL (detected via immunofluorescence staining) indicating that the cells in this group could develop mature axons. (b) In the presence of rapamycin (RAP), the axons surrounding the organoid were observed to be tangled. Thicker axons stained positive for both tubulin and NEFL, whereas thinner axons within the organoid stained positive for only tubulin, suggesting that many axons in this group were still immature. (c) In the presence of dexamethasone (DEX), axons were short and thin and most stained only for tubulin, indicating that most axons in this group were immature. (d) Cells stained positive for HuD in the control group also exhibited long and dense dendrites that (g) stained positive for MAP2. (e, f) RAP-treated and DEX-treated organoids showing cells staining positive for HuD. (h, i) RAP- and DEX-treated organoids showing cells staining positive for MAP2. Proliferative cells in (j) control, (k) RAP, and (l) DEX groups stained positive for Ki67. CTRL: control group; RAP: rapamycin-treatment group; DEX: dexamethasone-treatment group.

After attaching to coverslips, cells from retinal organoids migrated out from the organoid into the surrounding area (Figure 2(a)–(c)); following this, these cells were also found to develop axons. In the absence of immunosuppressants (control group), these axons were observed to be long and radial; furthermore, the cells from organoids in the control group were observed to migrate for longer distances than those in organoids treated with either RAP or DEX (Figure 2(d)). In addition, RAP treatment resulted in the formation of shorter axons, which also tended to be more intertwined (Figure 2(e)), whereas DEX treatment resulted in the formation of shorter and thinner axons (Figure 2(f)). Immunofluorescence staining further showed that in the control group, the majority of the organoid cells expressed HuD, tubulin, NEFL, and MAP2 (a marker of dendrites) (Figure 3(a), (d), and (g)), indicating that after attachment, these cells continued at RGC differentiation. The axons of these cells in the control group were also long and stained positive for both tubulin and NEFL. Taken together, these results suggest that the axons and dendrites developed by cells in the organoids of the control group were relatively mature. In the RAP- and DEX-treated groups, many cells from the organoids also expressed HuD, tubulin, NEFL, and MAP2 (Figure 3(b), (c), (e), (f), (h), and (i)), although many of them were morphologically different from cells in the control group. Axons in the RAP-treatment group were found to stain positive for both tubulin and NEFL (indicating that they were relatively mature), whereas, in the DEX-treatment group, axons only stained positive for tubulin. In addition, the dendrites in both RAP- and DEX-treatment groups were shorter and fewer compared to those in the control group. Further, the presence of Ki67-positive cells (indicating cells in the mitotic cycle) also suggested that cells in the organoids were capable of cell division with or without immunosuppressants. However, treatment with RAP and DEX appeared to decrease the numbers of proliferating cells (Figure 3(j)–(l)), which were observed to be increased in the control group. In all, our results indicate that even in the presence of the immunosuppressants RAP and DEX, most cells in retinal organoids can survive and differentiate into RGCs; however, the morphological characteristics of these cells were different from those of cells in the control group, and their proliferation was also decreased.

### 3.3. Immunosuppressants Do Not Affect RNA Expression Patterns or Retinal Ganglion Cell Proportions in Retinal Organoids

**Figure 4:**
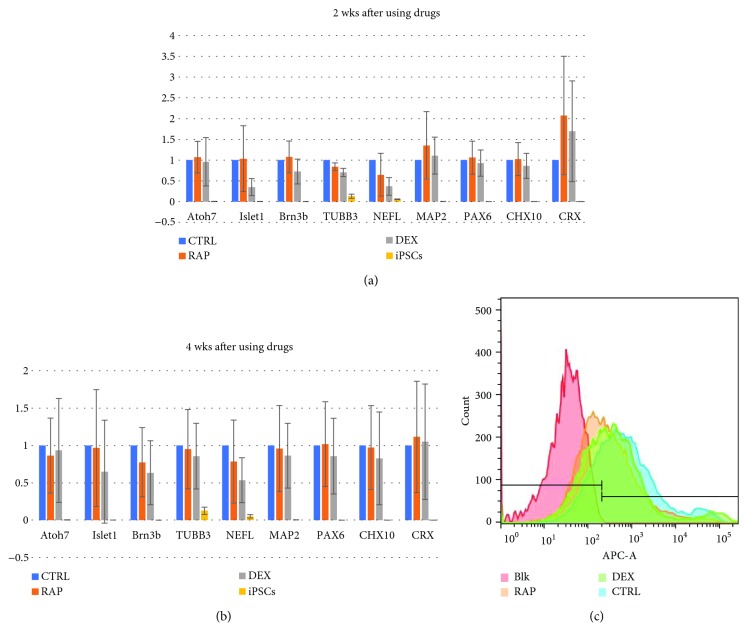
Comparisons of different marker expression profiles among the control, rapamycin- (RAP-) treated, and dexamethasone- (DEX-) treated groups. Two weeks after immunosuppressant treatment, (a) cells in the retinal organoids treated with RAP expressed higher levels of the retinal ganglion cell (RGC) markers Atoh7, Islet1, and Brn3b, the dendrite marker MAP2, the retinal progenitor marker PAX6 and CHX10, and photoreceptor marker CRX, compared to levels in the control group. These cells also expressed lower levels of the axon markers TUBB3 and NEFL than cells in the control group. In DEX-treatment group cells, dendrite, retinal progenitor, and photoreceptor markers (MAP2, CHX10, and CRX) were upregulated compared to levels in the control group. (b) However, 4 weeks after immunosuppressant treatment, both RAP- and DEX-treated cells showed higher expression levels of only PAX6 and CRX as compared to those in the control group. (c) Representative histogram of flow cytometry results. Each wave represented one group; the proportion of each group was calculated by the Blk group. There were 79.87 ± 5.92% of the RGCs in the control group, 60.97 ± 7.36% in the RAP group, and 63.6 ± 10.35% in the DEX group. CTRL: control group; RAP: rapamycin-treatment group; DEX: dexamethasone-treatment group; iPSCs: negative control group in RT-PCR; Blk: blank control group in flow cytometry.

The transcription of all investigated genes (except *TUBB3* and *NEFL*) was found to be upregulated 2 weeks after RAP treatment. Further, expression in the DEX-treatment group was lower than that in the RAP-treatment group. Four weeks after treatment with RAP and DEX, the expression levels of all genes were observed to be downregulated (except *CRX* in both groups and *PAX6* in the RAP-treatment group), probably due to the lack of neurotrophy. However, none of the differences between the RAP- and DEX-treatment groups were found to be statistically significant (Figure 4, *P* > 0.05). We also used a membrane surface antibody, CD90 [21, 22], to analyze the proportions of RGCs in the control, RAP, and DEX groups; these were found to be 79.87 ± 5.92%, 60.97 ± 7.36%, and 63.6 ± 10.35%, for the control, RAP-, and DEX-treatment groups, respectively; however, the differences among three groups were not statistically significant (*P* = 0.06).

### 3.4. Post-Operative Follow-Up after Transplantation of Retinal Organoids

**Figure 5:**
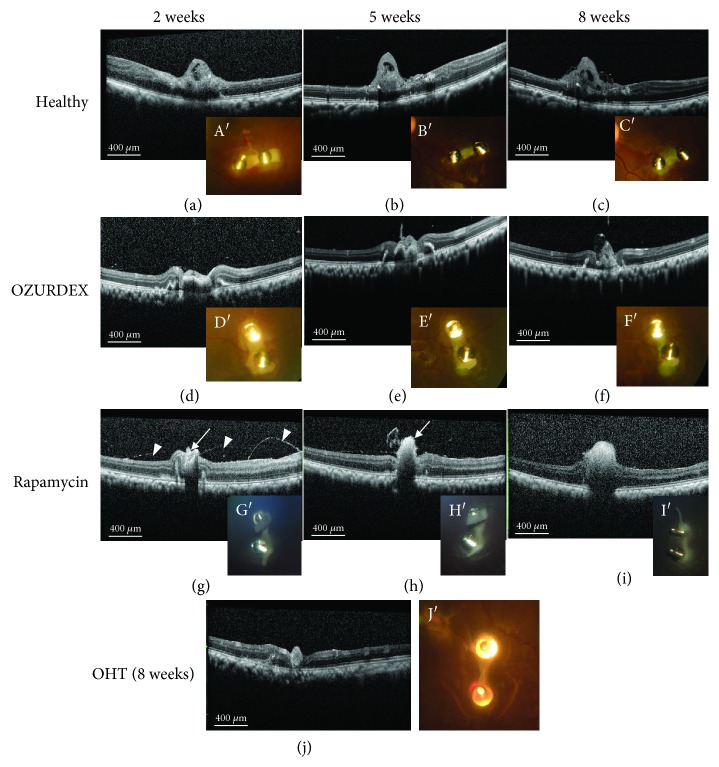
Changes in the retinal organoid after transplantation. In the healthy eye (transplanted with a retinal organoid, but receiving no immunosuppressive treatment), the transplanted retinal organoid sheet was clearly seen in the fundus photo. (A′) In the early post-transplantation stage (2 weeks), the sheet was white. (B′, C′) The green fluorescence in retinal organoid cells became more apparent at 5 and 8 weeks. Furthermore, the growth of cells in the retinal organoid was also apparent over a span of 1 week. (a–c) Optical coherence tomography (OCT) scanning showed that the organoid could be detected as a moderately reflective tissue above the host retina; OCT scanning also showed the growth of the organoid tissue. (a A′) Slight vitreous opacity was observed in the first 2 weeks after transplantation, but (b B′, c C′) the vitreous body soon became clear and remained so until the end of 8 weeks post-transplantation. (d D′–f F′) In the eye with ocular hypertension (OHT) transplanted with a retinal organoid and treated with dexamethasone (Oz-eye), the retinal organoid sheet was clearly visible and only slight vitreous opacity was observed in the first 2 weeks after transplantation. (g) In the OHT eye transplanted with a retinal organoid and treated with rapamycin (RAP-eye), the moderately reflective retinal organoid was only visible in the first two weeks post-transplantation (straight arrow), and highly reflective filaments were visible (arrowheads). The transplantation area only contained a patch of highly reflective tissue ((h), straight arrow) at 4 weeks. (g G′–i I′) The RAP-eye also exhibited intermediate levels of vitreous opacity 2, 5, and 8 weeks after transplantation. In the OHT-eye, which was transplanted with a retinal organoid but received no immunosuppressive treatment, severe vitreous opacity was observed and no fundus photos or OCT scans were carried out until week 8 post-transplantation. (j) At week 8 post-transplantation, OCT scanning of the OHT-eye indicated the presence of highly reflective tissue at the transplantation site, and (J′) a pale image of the organoid was visible in the fundus photo.

In the first 2 weeks after implantation, fundus photos and OCT scans indicated the presence of slight vitreous opacity in the healthy eye and OHT eyes with the OZURDEX implant (Oz-eye) (Figures 5(a) A′ and 5(d) D′); however, after 2 weeks, the vitreous body turned clear and remained so until the end of 8 weeks (Figures 5(b) B′, 5(c) C′, 5(e) E′, and 5(f) F′). However, the OHT eye treated with RAP (RAP-eye) and the OHT eye without immunosuppressant treatment (OHT-eye) showed vitreous opacity. The vitreous opacity in the RAP-eye (Figure 5(g) G′–5(i) I′) was moderate, whereas, during the first 5 weeks after transplantation, that in the OHT-eye was severe; however, after 5 weeks, the vitreous body in the OHT-eye gradually cleared up (Figure 5(j) J′. OCT scanning revealed that the transplanted retinal organoids were clearly visible in the healthy eye and Oz-eye, whereas the organoids were observed to gradually disappear in the RAP-eye, and at the end of 8 weeks, highly reflective patches of tissue were observed at the transplantation sites in the RAP-eye and OHT-eye (Figure 5(j)J`). These tissues might be fibrotic in nature.

### 3.5. Survival and Differentiation of Organoid Cells after Transplantation

**Figure 6:**
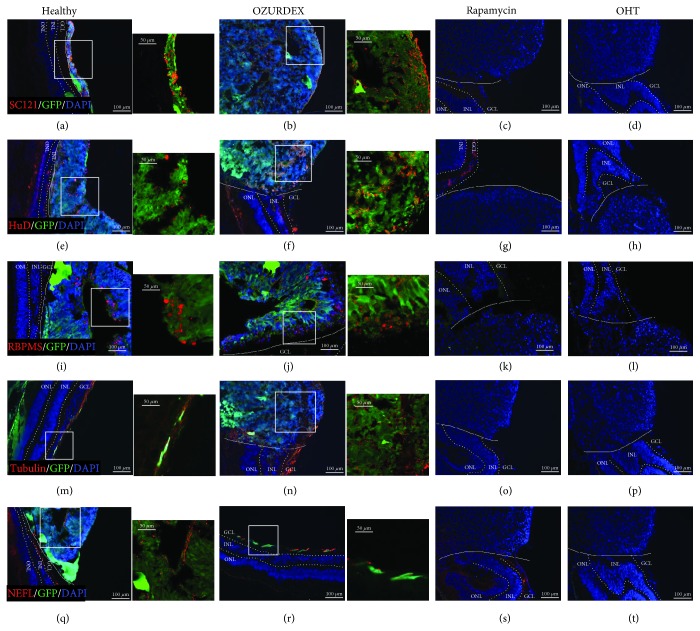
Survival and differentiation of retinal organoid cells as analyzed by immunofluorescence microscopy after transplantation. (a, b) The SC121-positive cells expressing green fluorescent protein (GFP), which were used for construction of the retinal organoids, were clearly visible in the healthy eye (transplanted with a retinal organoid, but receiving no immunosuppressive treatment) and the Oz-eye (eye with ocular hypertension (OHT) transplanted with a retinal organoid and treated with dexamethasone), indicating that cells from the transplanted organoids were alive in the host retina. However, in (c) the RAP-eye (OHT eye transplanted with a retinal organoid and treated with rapamycin) and (d) the OHT-eye (OHT eye transplanted with a retinal organoid, but receiving no immunosuppressive treatment), no SC121-positive or GFP-positive cells were detected. HuD- and RBPMS-positive retinal ganglion cells (RGCs) were also observed in (e, i) the healthy eye and (f, j) the Oz-eye. (m) Tubulin- and (q) NEFL-positive axons were observed within and near the transplants in the healthy eye. (n) Tubulin- and (r) NEFL-positive axons were observed within and near the transplants in the Oz-eye. In (c, g, k, o, s) the RAP-eye and (d, h, l, p, t) the OHT-eye, no cells or axons expressing these markers were detected. GCL: ganglion cell layer; INL: inner nuclear layer; ONL: outer nuclear layer.

Eight weeks after transplantation, the monkeys were euthanized and the transplanted eye tissues were analyzed by fluorescence microscopy. The organoid cells in the implants expressed green fluorescence protein (GFP) spontaneously and thus could be distinguished from the monkey eye tissues. Organoid cells expressing GFP were apparent in the healthy eye and Oz-eye, whereas no such cells were detected in the RAP-eye or OHT-eye. In addition, we also used SC121 antibodies, which specifically bind human cells, to test for the presence of cells from the transplanted organoids (Figure 6(a)–(d)) in the healthy eye and Oz-eye. Our immunofluorescence microscopy results further indicated that in the healthy eye and Oz-eye, cells in the implanted organoids still expressed molecular markers of RGCs (Figure 6(e), (f), (i), and (j)), and axons (Figure 6(m), (n), (q), and (r)) indicating that these cells might be capable of differentiating into RGCs after implantation.

### 3.6. Immune Responses to Implanted Organoids

**Figure 7:**
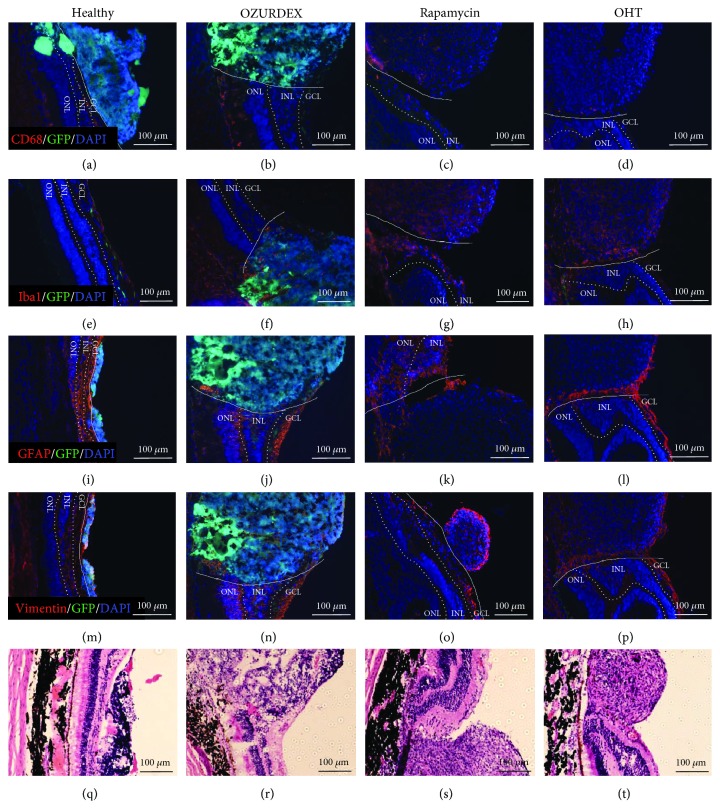
Immune responses after transplantation. (a, b, d) Few activated microglia (CD68-positive) were seen in the healthy eye (transplanted with a retinal organoid, but receiving no immunosuppressive treatment), Oz-eye (eye with ocular hypertension (OHT) transplanted with a retinal organoid and treated with dexamethasone), and OHT-eye (OHT eye transplanted with a retinal organoid, but receiving no immunosuppressive treatment). (c) However, many CD68-positive cells were seen in the RAP-eye (OHT eye transplanted with a retinal organoid and treated with rapamycin). (e) In the healthy eye, few microglia (Iba1-positive) were spread out within the inner retina. (f) Few microglia (Iba1-positive) were seen clustered near the transplant site in the Oz-eye. In the (g) RAP-eye and the (h) OHT-eye, large numbers of microglia (Iba1-positive) were found to be accumulated at the transplant sites. (i) GFAP- and (m) vimentin-positive Müller cells were distributed throughout the inner retina of the healthy eye. Similarly, (j) GFAP- and (n) vimentin-positive Müller cells were distributed throughout the inner retina of the Oz-eye. However, in the RAP-eye, (k) GFAP- and (o) vimentin-positive Müller cells were found surrounding the transplantation site. Similarly, in the OHT-eye, (l) GFAP- and (p) vimentin-positive Müller cells were found to surround the transplantation site. HE staining showed that in the (q) healthy eye and the (r) Oz-eye, the transplantation site had rosette-forming cell clusters with high nuclear-cytoplasmic ratios. In the (s) RAP-eye and the (t) OHT-eye, however, weakly basophilic cells were found to accumulate at the transplantation site. No obvious lymphocytes were detected around the transplantation site in four groups. GCL: ganglion cell layer; INL: inner nuclear layer; ONL: outer nuclear layer.

Since microglia are the major immune cells in the retina and Müller cells play an important role in gliosis [23], we used two microglial markers (CD68 and Iba1) and two Müller cell markers (GFAP and vimentin) to study the immune responses mounted against retinal organoid transplants. Eight weeks after transplantation, only a few CD68-positive (activated) microglial cells were detected at the transplantation site of the healthy eye, Oz-eye, and OHT-eye (Figure 7(a), (b), and (d)). In contrast, many activated microglia were found to be clustered at and near the transplantation site in the RAP-eye (Figure 7(c)). In addition, few Iba1-positive cells were observed in the inner retina of the healthy eye (Figure 7(e)) and the Oz-eye (Figure 7(f)), whereas large numbers of Iba1-positive cells were found to be clustered in and around the transplantation site in the RAP- and OHT-eyes (Figure 7(g) and (h)). Müller cells, which express GFAP and vimentin, and are usually distributed in the inner retina, were found to be present in this area in the healthy eye and Oz-eye (Figure 7(i), (j), (m), and (n)). However, these cells were found to surround the transplantation site in the RAP- and OHT-eyes (Figure 7(k), (l), (o), and (p)). HE staining indicated that no obvious lymphocytic aggregations were detectable at or near the implantation sites in any of the eyes. We also observed rosette-shaped cell clusters at the transplantation sites (Figure 7(q) and (r)) in the healthy eye and Oz-eye, indicating the establishment of neural stem cell clusters in these areas. However, in RAP- and OHT-eyes, fibrous nodule-like structures were observed at the transplantation sites (Figure 7(s) and (t)). Together, the results indicate that although no strong immune responses occurred in the healthy eye and the Oz-eye, the transplantation sites in the RAP- and OHT-eyes showed strong immunoreactive characteristics, with the RAP-eye showing active inflammatory reactions 8 weeks after transplantation.

## 4. Discussion

In this study, we compared the efficacy of two different immunosuppressants, RAP and DEX, in maintaining an epiretinal implant derived from hiPSCs in rhesus monkey eyes for up to 8 weeks. Our results indicate that the epiretinal transplant has a higher chance of survival with OZURDEX® treatment, a dexamethasone IVT implant, which effectively staved off an immune reaction against the retinal organoid transplant for 8 weeks. We even observed that cells from the retinal organoid transplant could survive and differentiate into RGCs in the DEX-treatment group in vitro. However, as we only used one monkey for each situation in this research, the results might be limited.

Since stem-cell therapy is a promising avenue for the treatment of retinal degenerative diseases; the necessity of using immunosuppressants to ensure transplantation success has been markedly debated. Lai et al. [24] demonstrated that immunosuppressive treatment can prolong the survival (to several months) of human fetal retinal pigment epithelium grafts in rabbits, whereas, without immunosuppression, these grafts fail to survive for even several weeks. However, the presence of the blood-retinal barrier in the eye often does not allow local concentrations of immunosuppressants to build up to effective levels when such agents are delivered via systemic administration routes. Furthermore, several reports indicate that systemic immunosuppressive treatment does not significantly improve the long-term survival of grafts in subretinal transplants [25–27]. To maintain effective local concentrations of immunosuppressants in the eye, the required systemic intake of such agents is likely to be so high that the side effects of such agents would eclipse the advantages of immune suppression to facilitate graft survival. To avoid such complications, we chose IVT injections and IVT implants as viable drug-delivery methods in this study. IVT delivery systems enable the maintenance of high concentrations of required immunosuppressants in the eye, while systemic levels of these agents remain very low, which also lowers the side effects associated with these drugs. However, Abud et al. [28] reported that single IVT doses of RAP and DEX were insufficient to improve the survival of subretinal allotransplants; this was likely due to the relatively short half-lives of DEX (~5.5 h) and RAP (~7 days) within the vitreous cavity. Therefore, we chose to use a sustained-release delivery system for DEX and repeated IVT injections (once every week) of RAP to maintain effective concentrations of these immunosuppressants at the transplantation site.

Due to its anti-inflammatory [29] and neuroprotective [13] effects, RAP was expected to be an ideal immunosuppressant for protecting stem-cell transplants; however, the results of our study indicate that RAP treatment did not sufficiently suppress inflammatory reactions in the eye, and because of this, the retinal organoid transplants failed to survive. It is possible that the RAP concentration used in this study (20 *μ*g/ml) might not have been appropriate for either effective immunosuppression or neuroprotection. Although a phase 2 clinical trial for RAP use in uveitis [30] indicated that concentrations of RAP up to 440 *μ*g per human eye (~98 *μ*g/ml) are safe and useful for the treatment of noninfectious uveitis, cells in the retinal organoids were incapable of surviving such high levels. The conflicting requirements of RAP concentrations for immune suppression and retinal organoid cell survival, therefore, render RAP an ineffective immunosuppressant for epiretinal implants.

## 5. Conclusions

Our study clearly indicates that immunosuppressants are necessary for transplant survival in epiretinal transplantation procedures involving retinal organoids and stem cells. We have also found that a single IVT implant (OZURDEX®) can provide effective immunosuppression to allow the survival of xenografts for up to 8 weeks after the epiretinal transplantation procedure. However, further investigations to determine how long immunosuppressive treatments are required for effective transplant integration are needed. Furthermore, controlled release formulations of other corticosteroids such as Retisert [31] and Iluvien [32] (which release immunosuppressants over longer durations of time) and other immunosuppressants such as tacrolimus, cyclosporin, and methotrexate, as well as combinations of immunomodulators, immunosuppressants, and neurotrophins such as BDNF (brain-derived neurotrophic factor) or CNTF (ciliary neurotrophic factor), must also be explored for use in epiretinal or vitreous transplantation procedures. In addition, since the side effects of intraocular corticosteroid administration also include glaucoma and cataract formation, more investigations involving routine IOP monitoring and post-operative follow-ups [33] to record the side effects of local corticosteroid treatment after transplantation are necessary.

## Figures and Tables

**Table 1:** Primers.

Gene	Forward (5′-3′)	Reverse (5′-3′)
Actin	GCGAGAAGATGACCCAGATC	CCAGTGGTACGGCCAGAGG
Atoh7	GGCGACACAGGACAATCTTTA	TTCCGGCAGCTCCGTTTTC
Islet1	ATGACAAAACTAATATCCAGGGG	CTGAAAAATTGACCAGTTGCTG
Brn3b	CAAGCAGCGACGCATCAAG	GGGTTTGAGCGCGATCATATT
TUBB3	GGCCAAGGGTCACTACACG	GCAGTCGCAGTTTTCACACTC
NEFL	ATGAGTTCCTTCAGCTACGAGC	CTGGGCATCAACGATCCAGA
MAP2	GGGCCTTTCTTTGAAATCTAGTTT	CAAATGTGGCTCTCTGAAGAACA
PAX6	TGGGCAGGTATTACGAGACTG	ACTCCCGCTTATACTGGGCTA
CHX10	CTCGTGATATGCTGCTTGTG	GCCTGTGGCTTCGTAGATG
CRX	GCCCCACTATTCTGTCAACG	GTCTGGGTACTGGGTCTTGG

**Table 2:** Rapamycin and dexamethasone suppressed microglia.

Drugs (concentration)	Dexamethasone (170 *μ*g/ml)	Rapamycin (20 *μ*g/ml)	Activated microglia
Inhibiting rate (%)^∗^	48.74 ± 0.04	57.22 ± 0.18	0
TNF-*α* (pg/ml)^∗∗^	605.51 ± 248.42	437.74 ± 102.13	2247.79 ± 257.57
NO (*μ*M)^∗∗^	20.46 ± 3.11	14.89 ± 2.10	39.70 ± 1.76

^∗^No statistical difference was detected between DEX and RAP. ^∗∗^Statistical differences were detected in three groups, but the difference between DEX and RAP was not statistically significant.

## Data Availability

The figure data used to support the findings of this study are included within the article.

## Supplementary Materials

Supplementary 1 Supplementary figure 1: the single fluorescent channel for Figure 7(a)–(d). Activated microglia (CD68-positive) in different situations after 8 weeks transplantation. Few activated microglia were seen in the healthy eye (transplanted with a retinal organoid, but receiving no immunosuppressive treatment), Oz-eye (eye with ocular hypertension (OHT) transplanted with a retinal organoid and treated with dexamethasone), and OHT-eye (OHT eye transplanted with a retinal organoid, but receiving no immunosuppressive treatment). (C) However, many CD68-positive cells were seen in the RAP-eye (OHT eye transplanted with a retinal organoid and treated with rapamycin).

Supplementary 2 Supplementary figure 2: the single fluorescent channel for Figure 7(e)–(h). Microglia (Iba1-positive) in different situations after 8 weeks transplantation. In the healthy eye, few microglia were spread out within the inner retina. Few microglia were seen clustered near the transplant site in the Oz-eye. In the RAP-eye and the OHT-eye, large numbers of microglia were found to be accumulated at the transplant sites.

Supplementary 3 Supplementary figure 3: the single fluorescent channel for Figure 7(i)–(l). Müller cells in different situations after 8 weeks transplantation. GFAP-positive Müller cells were distributed throughout the inner retina of the healthy eye. Similarly, Müller cells were distributed throughout the inner retina of the Oz-eye. However, in the RAP-eye, Müller cells were found surrounding the transplantation site. Similarly, in the OHT-eye, Müller cells were found to surround the transplantation site.

Supplementary 4 Supplementary figure 4: the single fluorescent channel for Figure 7(m)–(p). Müller cells in different situations after 8 weeks transplantation. Vimentin-positive Müller cells were distributed throughout the inner retina of the healthy eye. Similarly, Müller cells were distributed throughout the inner retina of the Oz-eye. However, in the RAP-eye, Müller cells were found surrounding the transplantation site. Similarly, in the OHT-eye, Müller cells were found to surround the transplantation site in different situations after 8 weeks transplantation.
