# Supplementary material for: Dexamethasone Provides Effective Immunosuppression for Improved Survival of Retinal Organoids after Epiretinal Transplantation
Source: Stem Cells Int. 2019 Jul 25;2019:7148032. doi: 10.1155/2019/7148032 (PMC6683795; doi:10.1155/2019/7148032)
Supplement: Supplementary 1 — Supplementary figure 1: the single fluorescent channel for Figure 7(a)–(d). Activated microglia (CD68-positive) in different situations after 8 weeks transplantation. Few activated microglia were seen in the healthy eye (transplanted with a retinal organoid, but receiving no immunosuppressive treatment), Oz-eye (eye with ocular hypertension (OHT) transplanted with a retinal organoid and treated with dexamethasone), and OHT-eye (OHT eye transplanted with a retinal organoid, but receiving no immunosuppressive treatment). (C) However, many CD68-positive cells were seen in the RAP-eye (OHT eye transplanted with a retinal organoid and treated with rapamycin). [file 7148032.f1.docx]

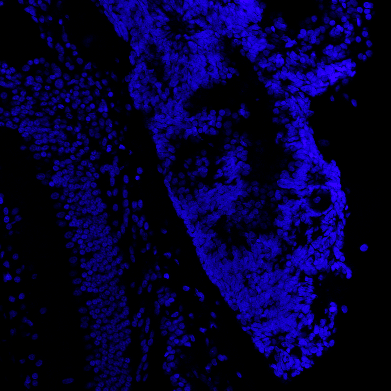

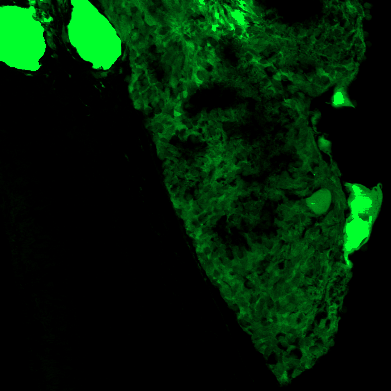

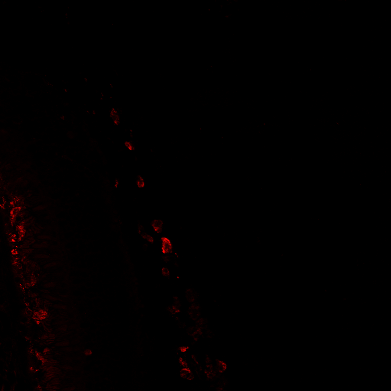

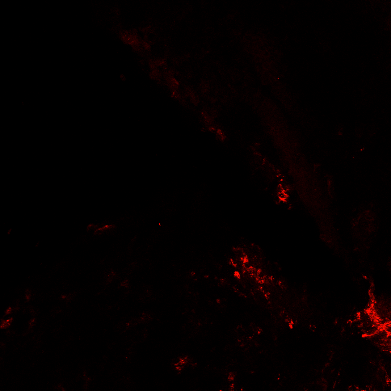

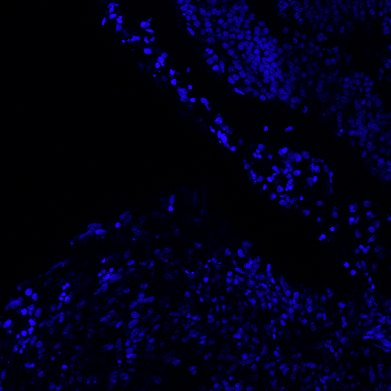

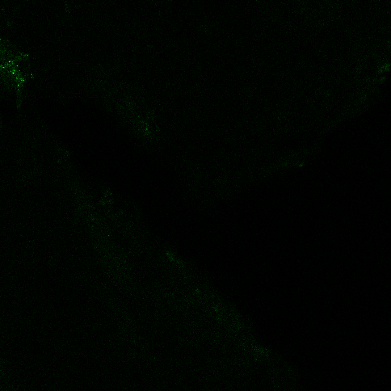

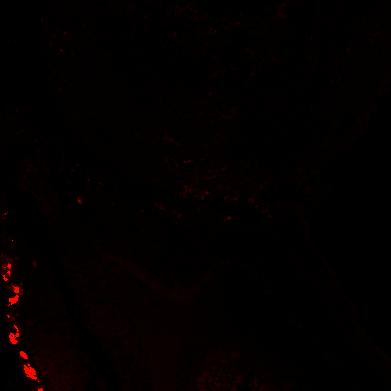

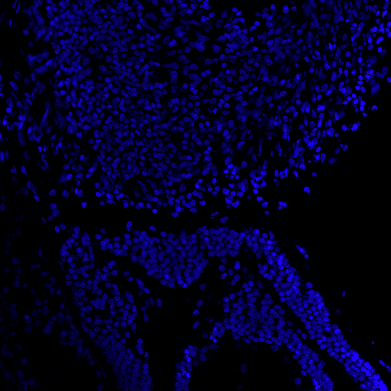

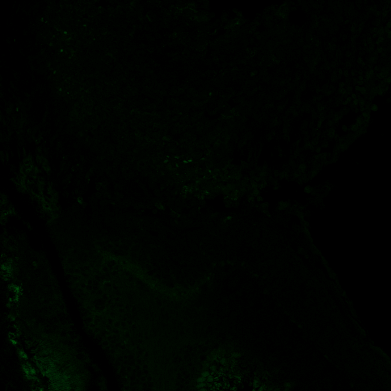

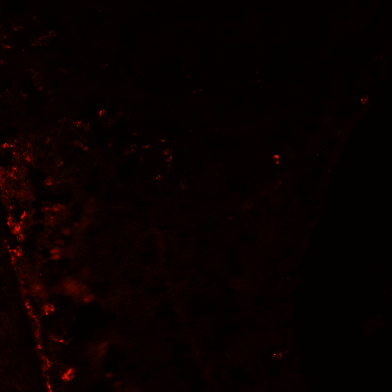

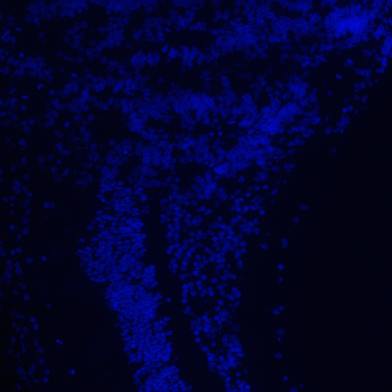

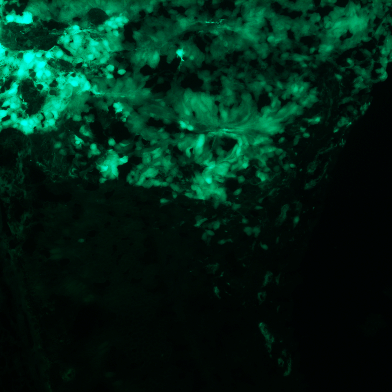

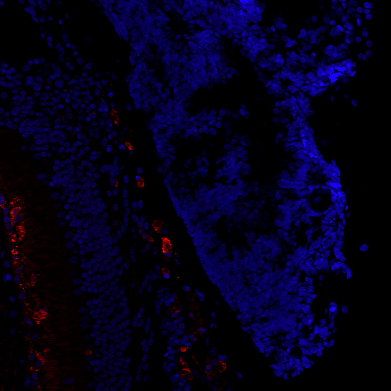

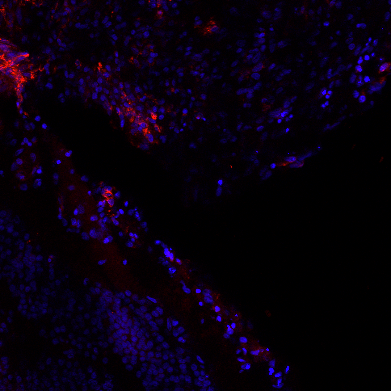

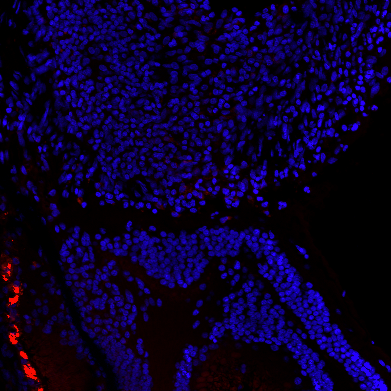

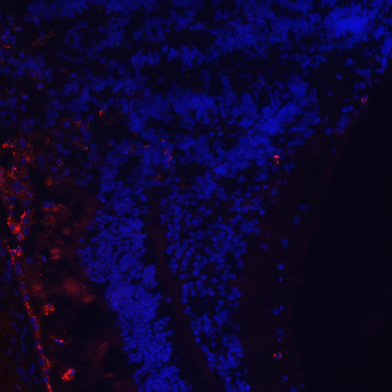

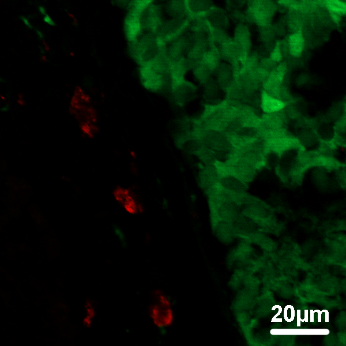

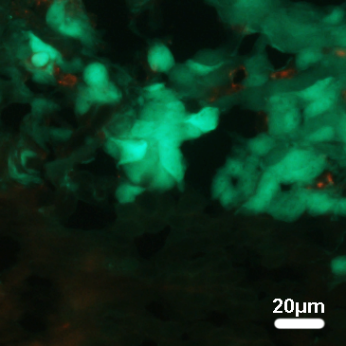

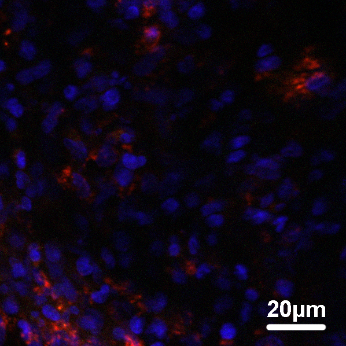

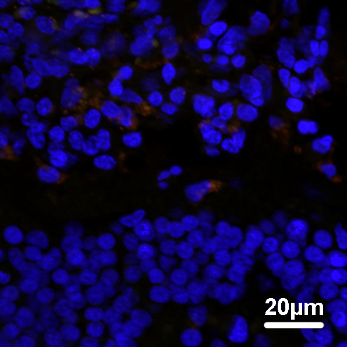


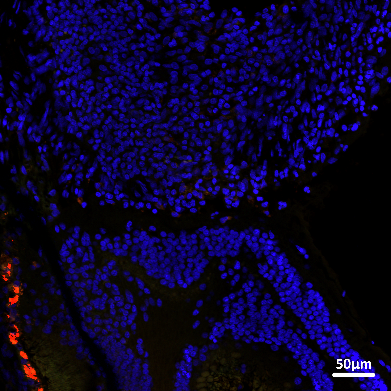

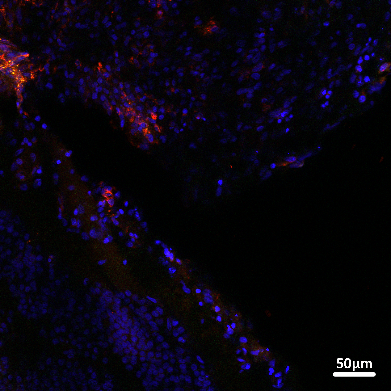

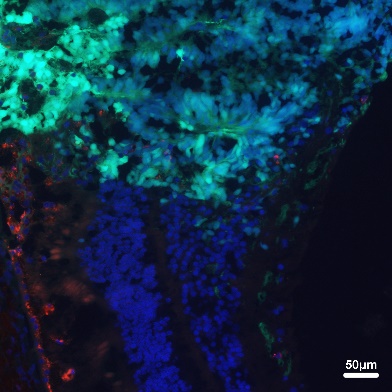

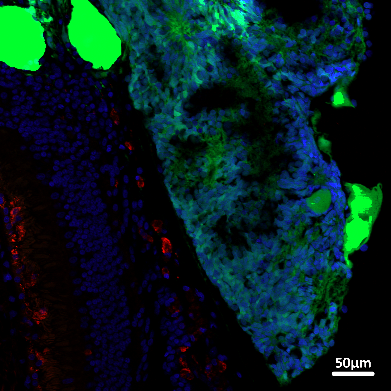


Healthy

OZURDEX

Rapamycin

OHT

**CD68**

**DAPI**

**GFP**

Supplementary figure 1 The single fluorescent channel for Figure7 A-D

Activated microglia (CD68-positive) in different situation after 8-weeks transplantation. Few activated microglia were seen in the healthy eye (transplanted with a retinal organoid, but receiving no immunosuppressive treatment), Oz-eye (eye with ocular hypertension (OHT) transplanted with a retinal organoid and treated with dexamethasone), and OHT-eye (OHT eye transplanted with a retinal organoid, but receiving no immunosuppressive treatment). (C) However, many CD68-positive cells were seen in the RAP-eye (OHT eye transplanted with a retinal organoid and treated with rapamycin).
