# Supplementary material for: Dexamethasone Provides Effective Immunosuppression for Improved Survival of Retinal Organoids after Epiretinal Transplantation
Source: Stem Cells Int. 2019 Jul 25;2019:7148032. doi: 10.1155/2019/7148032 (PMC6683795; doi:10.1155/2019/7148032)
Supplement: Supplementary 2 — Supplementary figure 2: the single fluorescent channel for Figure 7(e)–(h). Microglia (Iba1-positive) in different situations after 8 weeks transplantation. In the healthy eye, few microglia were spread out within the inner retina. Few microglia were seen clustered near the transplant site in the Oz-eye. In the RAP-eye and the OHT-eye, large numbers of microglia were found to be accumulated at the transplant sites. [file 7148032.f2.docx]

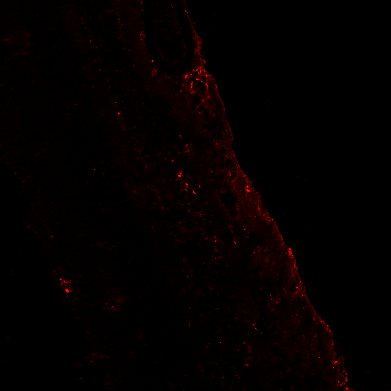

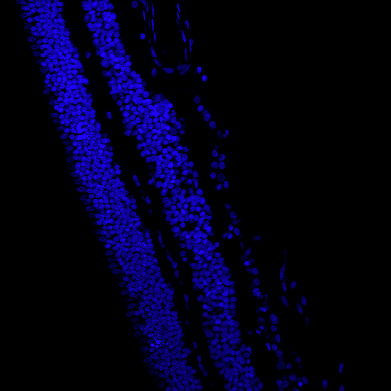

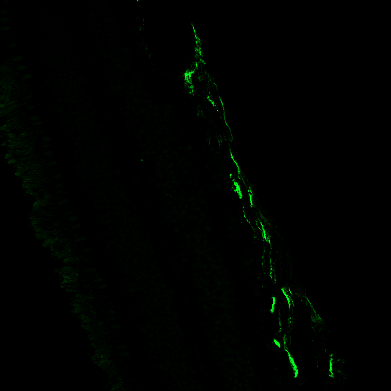

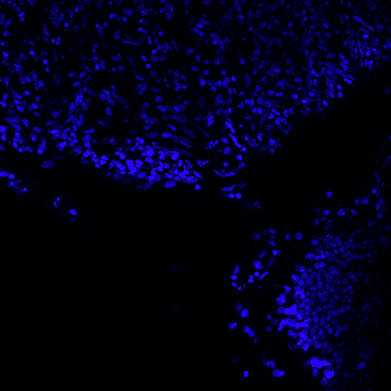

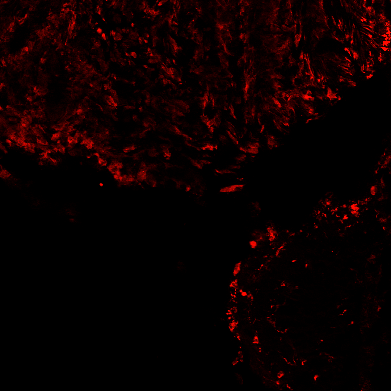

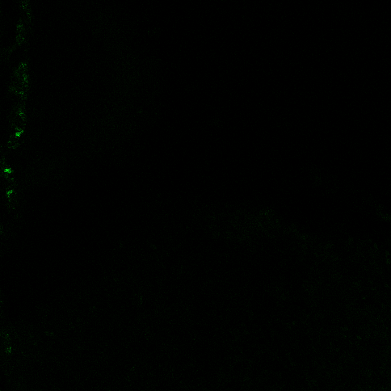

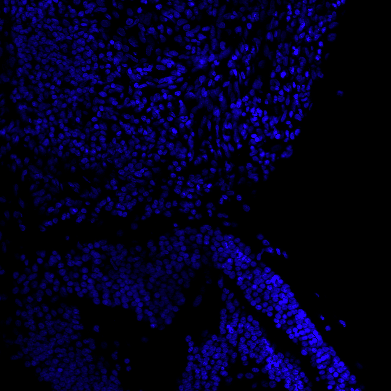

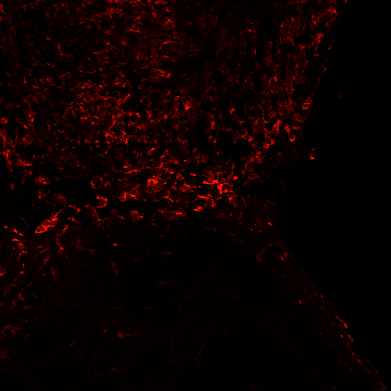

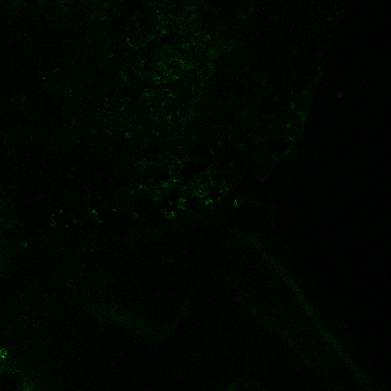

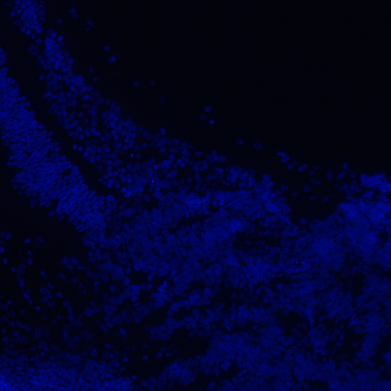

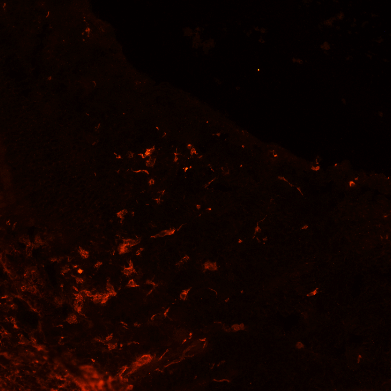

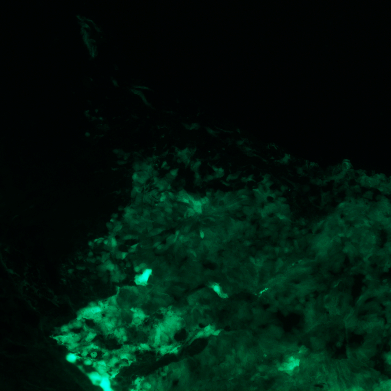

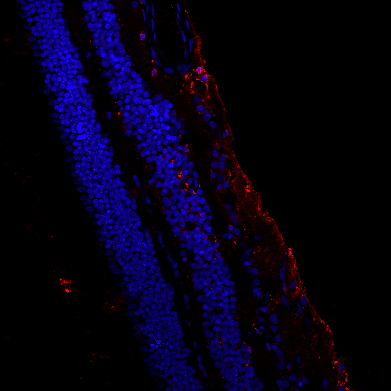

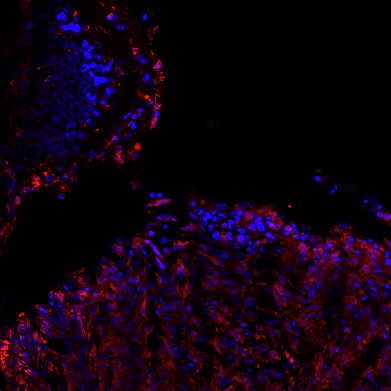

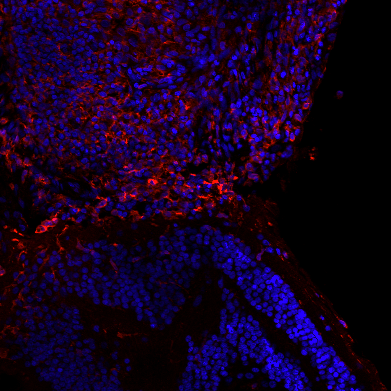

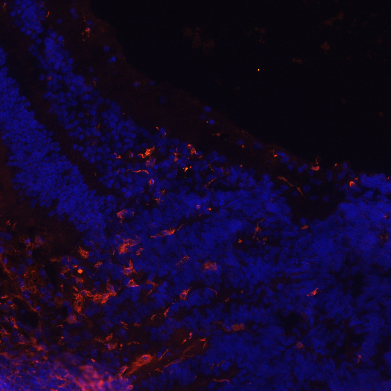

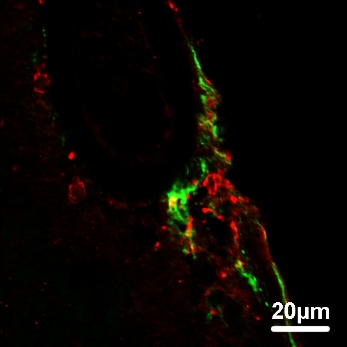

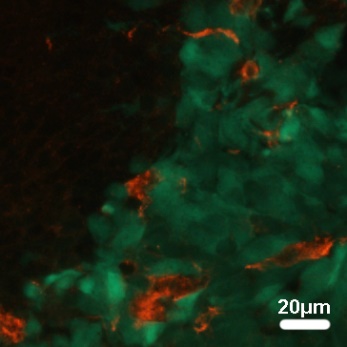

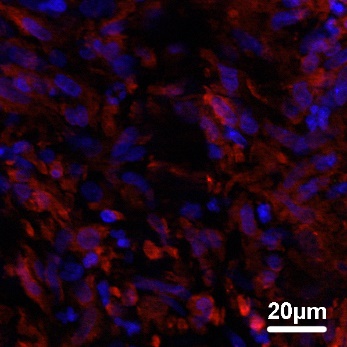

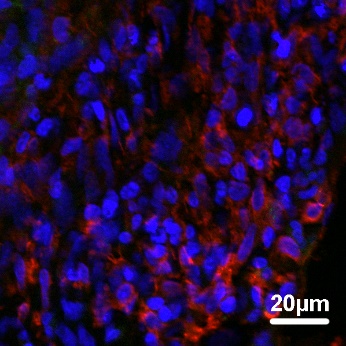


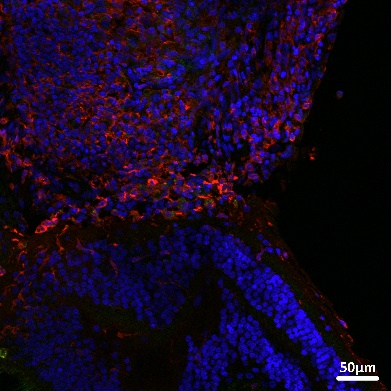

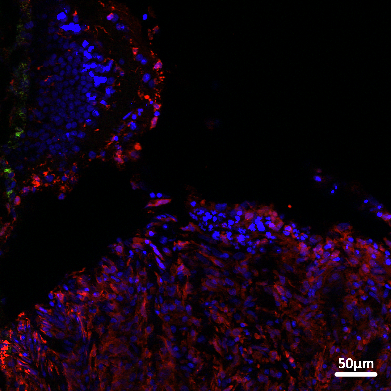

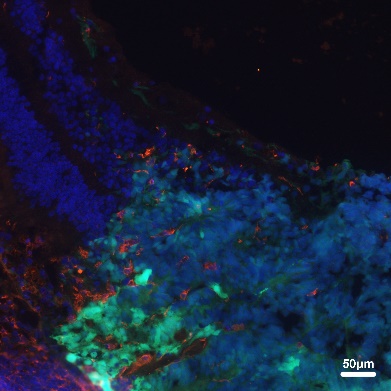

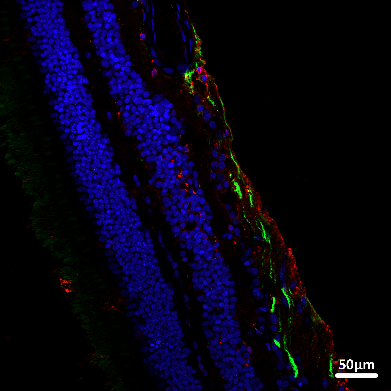


**Iba1**

**DAPI**

**GFP**

Healthy

OZURDEX

Rapamycin

OHT

Supplementary figure 2 The single fluorescent channel for Figure7 E-H

Microglia (Iba1-positive) in different situation after 8-weeks transplantation. In the healthy eye, few microglia were spread out within the inner retina. Few microglia were seen clustered near the transplant site in the Oz-eye. In the RAP-eye and the OHT-eye, large numbers of microglia were found to be accumulated at the transplant sites.
