# Supplementary material for: Dexamethasone Provides Effective Immunosuppression for Improved Survival of Retinal Organoids after Epiretinal Transplantation
Source: Stem Cells Int. 2019 Jul 25;2019:7148032. doi: 10.1155/2019/7148032 (PMC6683795; doi:10.1155/2019/7148032)
Supplement: Supplementary 3 — Supplementary figure 3: the single fluorescent channel for Figure 7(i)–(l). Müller cells in different situations after 8 weeks transplantation. GFAP-positive Müller cells were distributed throughout the inner retina of the healthy eye. Similarly, Müller cells were distributed throughout the inner retina of the Oz-eye. However, in the RAP-eye, Müller cells were found surrounding the transplantation site. Similarly, in the OHT-eye, Müller cells were found to surround the transplantation site. [file 7148032.f3.docx]

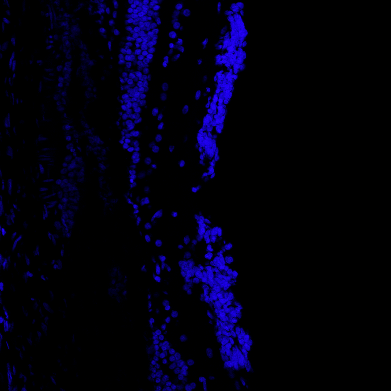

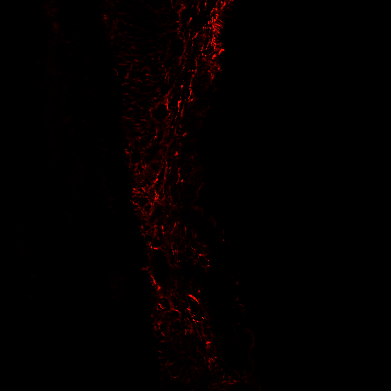

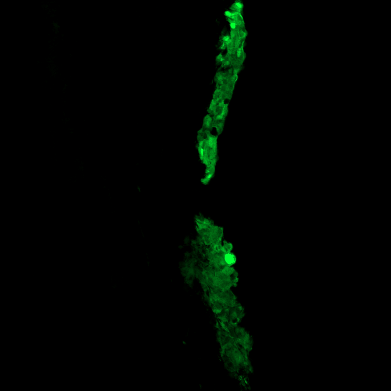

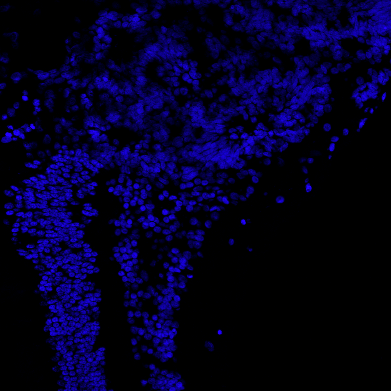

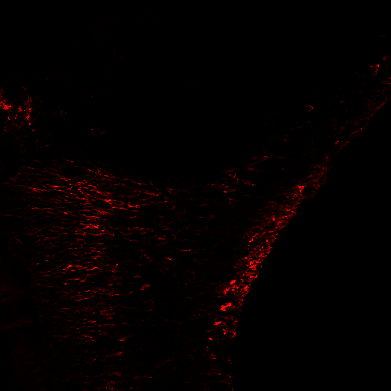

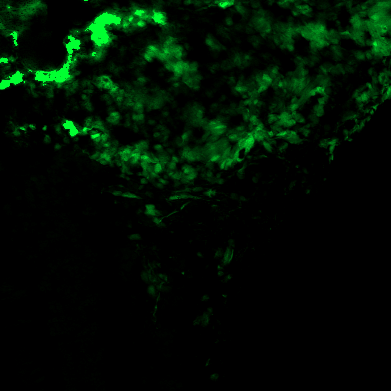

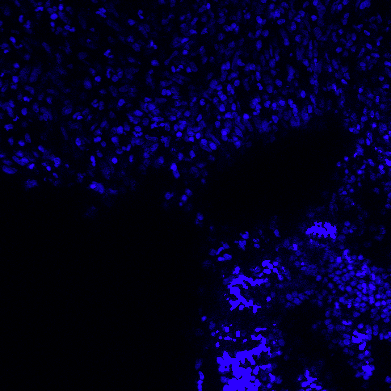

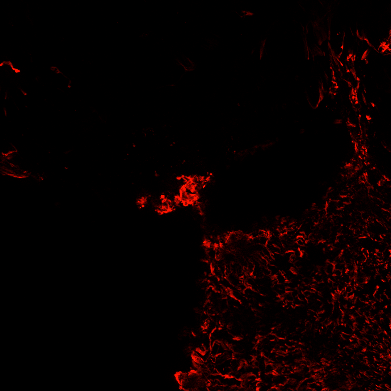

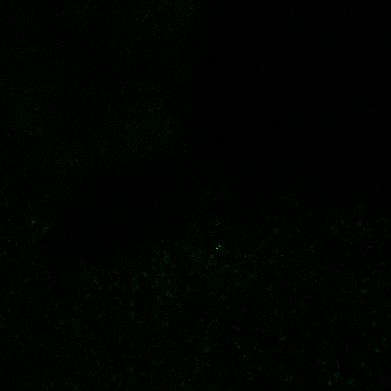

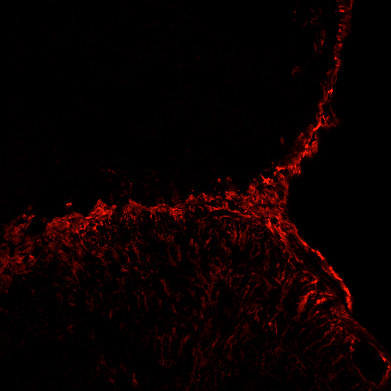

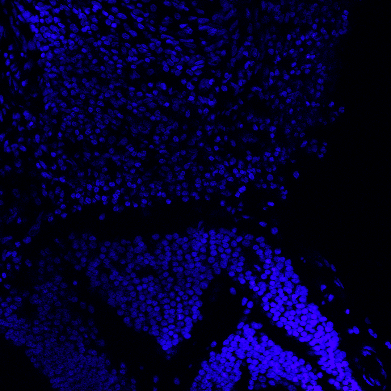

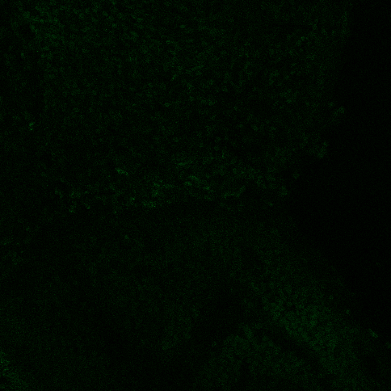

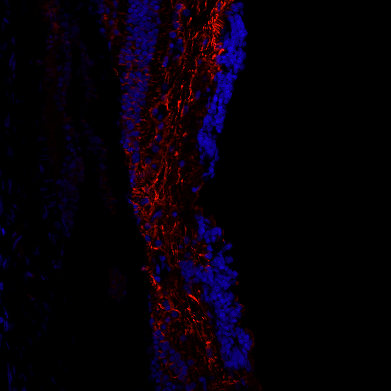

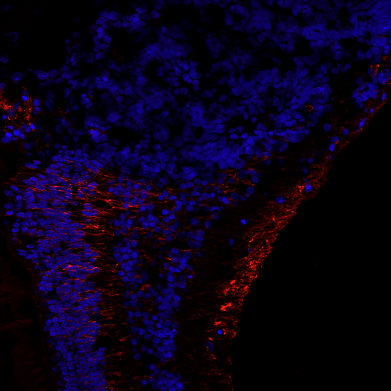

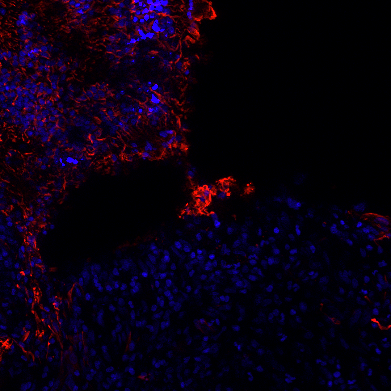

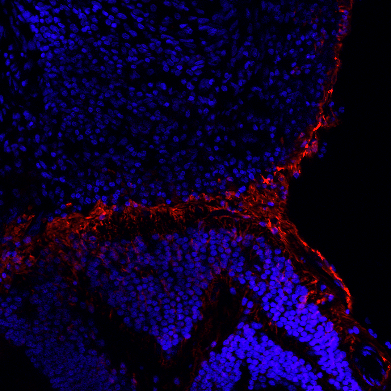

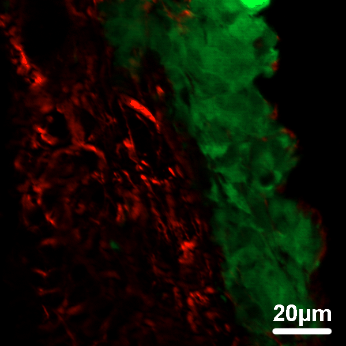

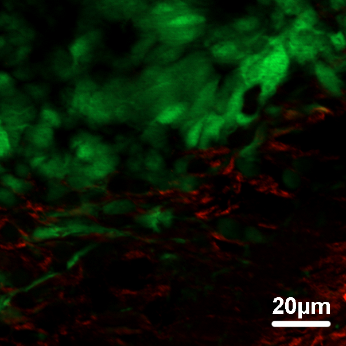

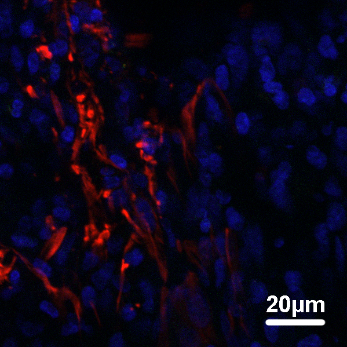

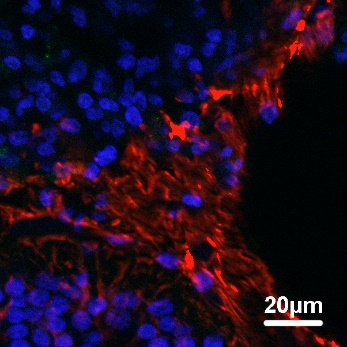


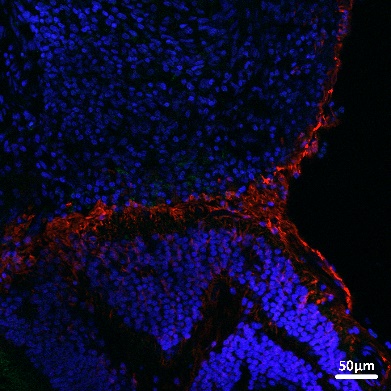

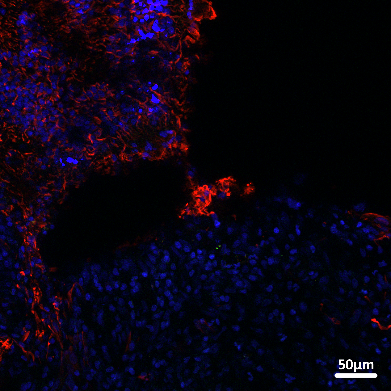

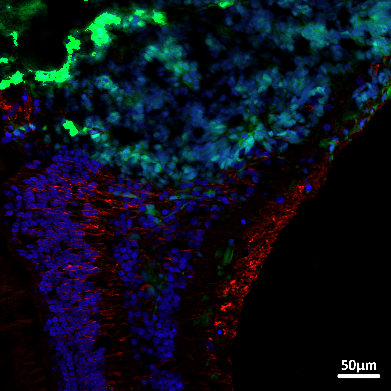

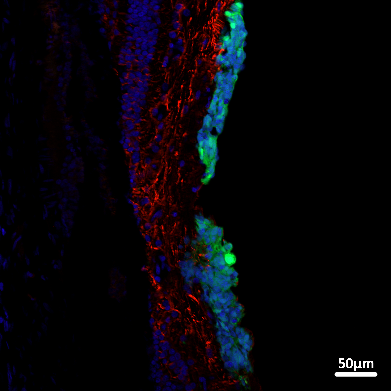


**GFAP**

**DAPI**

**GFP**

Healthy

OZURDEX

Rapamycin

OHT

Supplementary figure 3 The single fluorescent channel for Figure7 I-L

Müller cells in different situation after 8-weeks transplantation. GFAP-positive Müller cells were distributed throughout the inner retina of the healthy eye. Similarly, Müller cells were distributed throughout the inner retina of the Oz-eye. However, in the RAP-eye, Müller cells were found surrounding the transplantation site. Similarly, in the OHT-eye, Müller cells were found to surround the transplantation site.
