# Supplementary material for: Dexamethasone Provides Effective Immunosuppression for Improved Survival of Retinal Organoids after Epiretinal Transplantation
Source: Stem Cells Int. 2019 Jul 25;2019:7148032. doi: 10.1155/2019/7148032 (PMC6683795; doi:10.1155/2019/7148032)
Supplement: Supplementary 4 — Supplementary figure 4: the single fluorescent channel for Figure 7(m)–(p). Müller cells in different situations after 8 weeks transplantation. Vimentin-positive Müller cells were distributed throughout the inner retina of the healthy eye. Similarly, Müller cells were distributed throughout the inner retina of the Oz-eye. However, in the RAP-eye, Müller cells were found surrounding the transplantation site. Similarly, in the OHT-eye, Müller cells were found to surround the transplantation site in different situations after 8 weeks transplantation. [file 7148032.f4.docx]

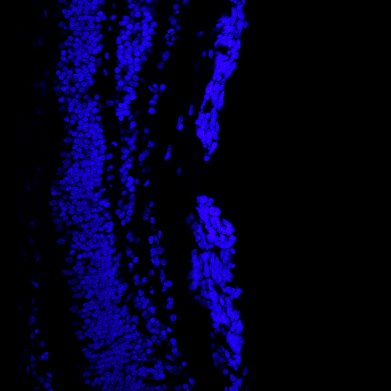

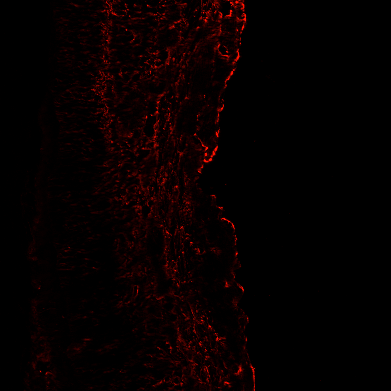

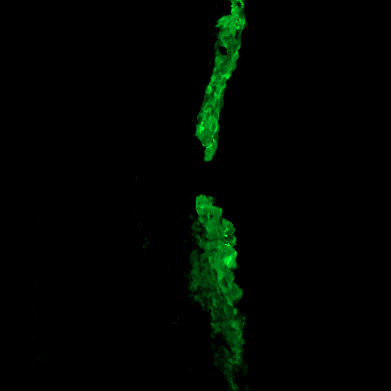

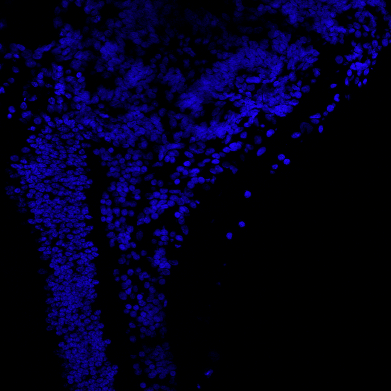

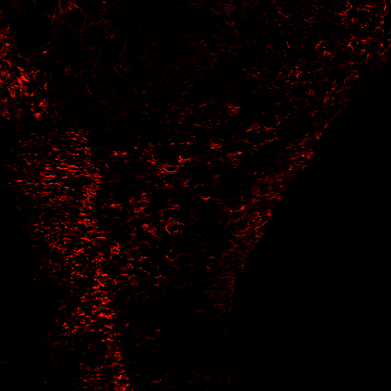

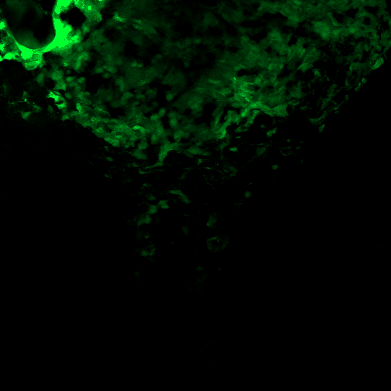

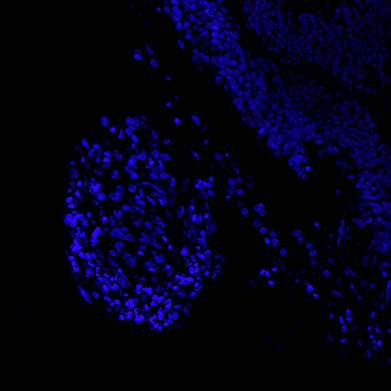

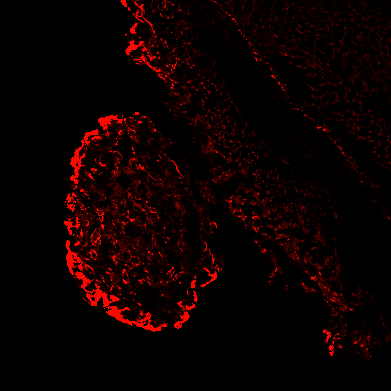

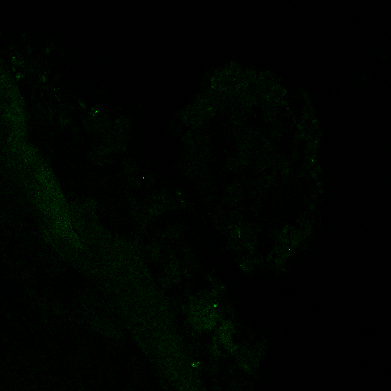

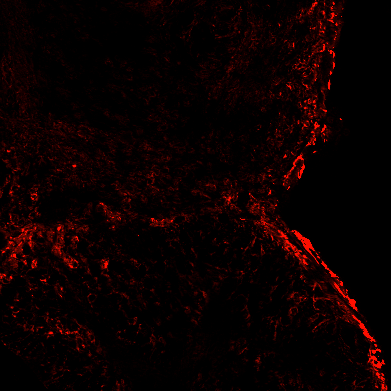

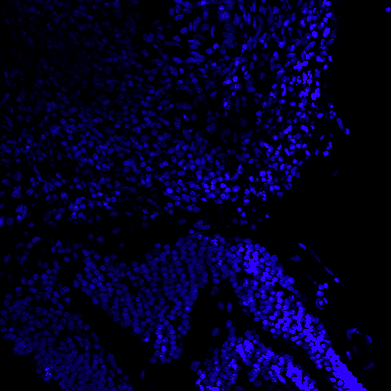

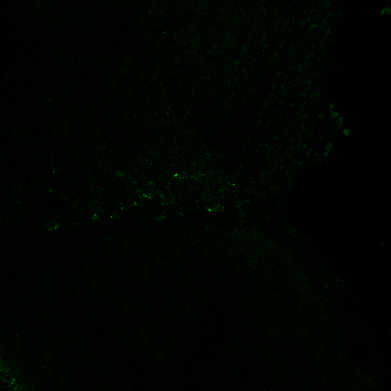

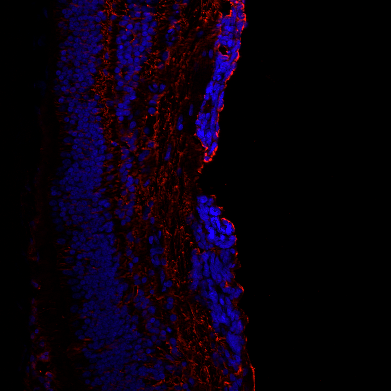

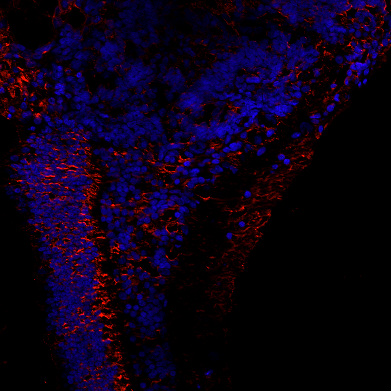

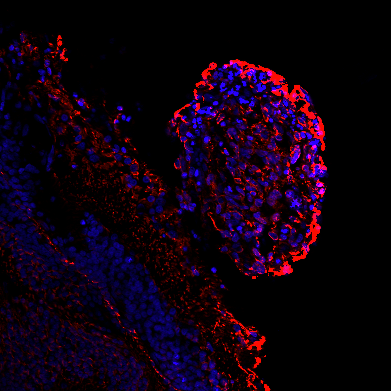

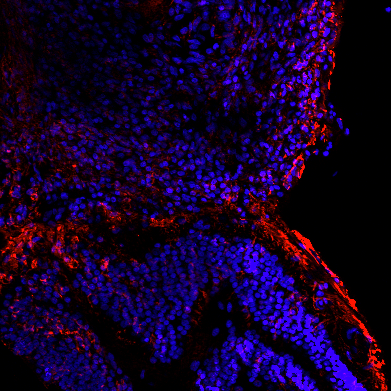

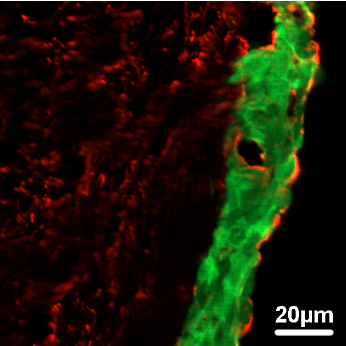

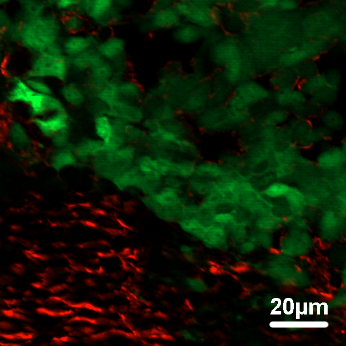

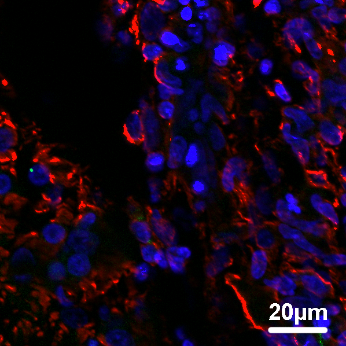

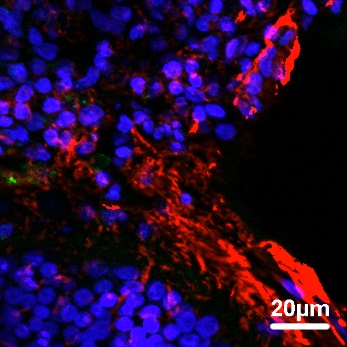


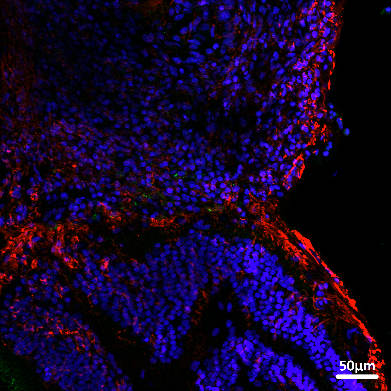

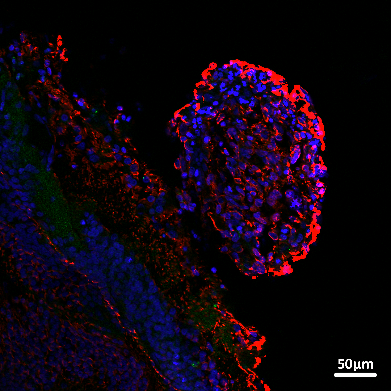

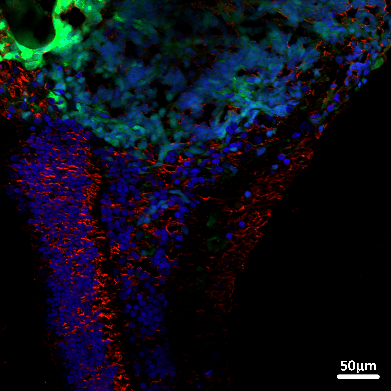

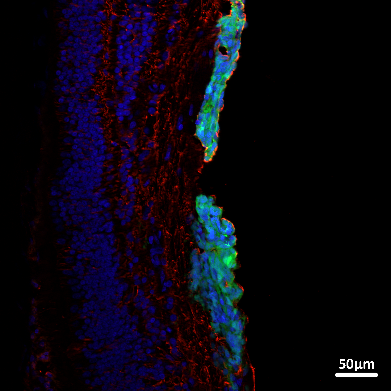


**vimentin**

**DAPI**

**GFP**

Healthy

OZURDEX

Rapamycin

OHT

Supplementary figure 4 The single fluorescent channel for Figure7 M-P

Müller cells in different situation after 8-weeks transplantation. vimentin-positive Müller cells were distributed throughout the inner retina of the healthy eye. Similarly, Müller cells were distributed throughout the inner retina of the Oz-eye. However, in the RAP-eye, Müller cells were found surrounding the transplantation site. Similarly, in the OHT-eye, Müller cells were found to surround the transplantation site.

in different situation after 8-weeks transplantation.
